# Development and Evaluation of a Colourimetric LAMP Assay for Field Detection of *Erysipelothrix rhusiopathiae* in Poultry Using a Rapid DNA Extraction Method

**DOI:** 10.1002/vms3.71100

**Published:** 2026-08-12

**Authors:** Seyed Ali Ghorashi, Jiongrui Huang, Peter Scott, Sameer D. Pant

**Affiliations:** ^1^ School of Agricultural Environmental and Veterinary Sciences, Charles Sturt University Wagga Wagga Australia; ^2^ Gulbali Institute Charles Sturt University Wagga Wagga Australia; ^3^ Scolexia Pty Ltd. Victoria Australia

**Keywords:** *Erysipelothrix rhusiopathiae*, LAMP, PCR, point‐of‐care assessment, rapid DNA extraction

## Abstract

**Background:**

Rapid and reliable diagnostic methods suitable for field use are needed for the detection of *Erysipelothrix rhusiopathiae* in poultry.

**Objectives:**

This study aims to develop and evaluate a colourimetric loop‐mediated isothermal amplification (LAMP) assay for the rapid detection of *E. rhusiopathiae* in conjunction with a rapid DNA extraction procedure suitable for field conditions.

**Methods:**

Specific primers targeting a conserved region of a putative polypeptide gene involved in the synthesis of capsular polysaccharides were used for LAMP and PCR. The specificity of the developed assays was evaluated using 13 unrelated bacterial strains. The efficacy of the assays was assessed using positive controls and 52 clinical samples collected from poultry farms experiencing erysipelas.

**Results:**

Both LAMP and PCR showed high sensitivity and specificity when compared with bacterial culture. LAMP and PCR achieved 100% sensitivity and specificity when combined with the rapid DNA extraction method. Using a commercial DNA extraction kit, LAMP maintained 100% sensitivity and specificity, whereas PCR showed 100% sensitivity and 97.8% specificity. The high correlation observed between bacterial culture and the LAMP assay demonstrated the effectiveness and reliability of the rapid DNA extraction method.

**Conclusions:**

The developed colourimetric LAMP assay provides a rapid, sensitive, and practical diagnostic tool for the detection of *E. rhusiopathiae* in poultry. The assay requires minimal instrumentation, enables straightforward visual interpretation of results, and is suitable for point‐of‐care testing and resource‐limited settings.

## Graphical abstract

This study presents a colourimetric LAMP assay combined with a rapid DNA extraction method for the field detection of *Erysipelothrix rhusiopathiae* in poultry. The workflow provides rapid and reliable diagnosis without specialised laboratory equipment, supporting on‐farm disease surveillance and outbreak management.

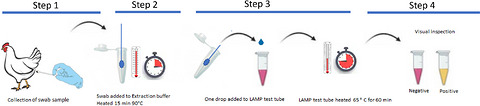

## 1 Introduction

*Erysipelothrix rhusiopathiae* is an important pathogen in poultry, where outbreaks of erysipelas can cause sudden mortality and production losses. Erysipelas is a global zoonotic disease that causes septicaemia in poultry and other animals, and affected birds often show lesions consistent with septicaemia (Eriksson 2019). Diagnosis usually relies on post‐mortem examination followed by the detection of *E. rhusiopathiae* using culture or PCR (Clark 2015; Zhao et al. 2023), which requires laboratory facilities and is not suitable for rapid, on‐farm decision‐making. This highlights the need for simple and rapid diagnostic methods that can be used in the field. Loop‐mediated isothermal amplification (LAMP) is a practical option for field diagnostics because it is rapid, specific, and does not require specialised equipment (Notomi et al. 2000). The outcomes of LAMP‐based diagnostic assays can be inferred based on changes in turbidity, fluorescence, or simply the colour of the reaction. LAMP assays where outcomes can simply be observed by the visualisation of colour changes (colourimetric LAMPs) are particularly suited for use in field for routine surveillance of pathogens (Calvert et al. 2017; Papadakis et al. 2021; Wang et al. 2021; Zhang et al. 2020). Colourimetric LAMP assays are particularly useful in low‐resource settings, as results can be interpreted visually without instruments. However, the major limitation to applying LAMP in the field is the need for a DNA extraction method that does not rely on laboratory instruments such as centrifuges.

Only one LAMP assay for *E. rhusiopathiae* has been published, and that study was performed on swine isolates using laboratory‐based DNA extraction and cultured colonies (Yamazaki et al. 2014). No colourimetric LAMP assay or rapid DNA extraction method has been evaluated for poultry samples collected during active outbreaks. As a result, there is currently no field‐ready molecular diagnostic workflow for erysipelas in poultry. The aim of this study was to develop and evaluate a rapid, colourimetric LAMP assay for detecting *E. rhusiopathiae* in poultry, together with a simple alkaline lysis DNA extraction method suitable for field use.

## 2 Material and Methods

### 2.1 *Erysipelothrix rhusiopathiae* Isolates and Clinical Samples

#### 2.1.1 Sample Collection

The development and evaluation of the LAMP assay involved three distinct sample sets. First, the *E. rhusiopathiae* vaccine strain, Eryvac, was used as a positive control for both LAMP and PCR assay development. Twelve swab samples from various chicken organs at a poultry farm experiencing erysipelas, along with 40 cloacal swab samples from a turkey flock at a commercial property with a history of erysipelas infection, were collected. This resulted in a total of 52 swabs used as clinical samples for assay development. However, the initial 12 clinical swabs underwent microbiological culturing before undergoing LAMP and PCR testing. In addition to the positive controls and clinical samples, 13 different bacterial species unrelated to *E. rhusiopathiae* (*Streptococcus equi* subsp. *zooepidemicus*, *Pasteurella multocida*, *Staphylococcus aureus*, *Escherichia coli*, *Salmonella typhimurium*, *Staphylococcus intermedius*, *Enterobacter cloacae*, *Klebsiella pneumoniae*, *Staphylococcus* sp., *Pseudomonas aeruginosa*, *Salmonella enteritidis*, *Mycoplasma gallisepticum*, and *Mycoplasma synoviae*) were obtained from the institutional veterinary diagnostic laboratory. These bacterial species were utilised to assess the test specificity during the LAMP assay development.

### 2.2 DNA Extraction

Two DNA extraction methods were used for the vaccine strain and all clinical samples. The first method used the Wizard Genomic DNA Purification Kit (Promega, Australia), and extraction was performed according to the manufacturer's instructions. The second method used the alkaline lysis DNA extraction approach (HotSHOT) described by Truett et al. (2000), which provides a rapid, low‐cost alternative suitable for field use. Although originally developed for mouse tissue, this method has since been applied to a range of sample types, including clinical swabs.

The commercial kit was used to extract DNA from the *E. rhusiopathiae* vaccine strain and 52 clinical swab samples. Furthermore, the *E. rhusiopathiae* vaccine was diluted in dH_2_O, and a single dose of the vaccine was applied to a cotton swab. Each of these vaccine‐loaded cotton swabs were subjected to DNA extraction separately, via both the alkaline lysis and commercial kit extraction protocols. Extracted DNA from all methods was quantified and assessed using a Nanodrop 2000 (Thermo Scientific, Australia) before use in PCR and LAMP assays.

### 2.3 LAMP and PCR Primers

**FIGURE 1:**
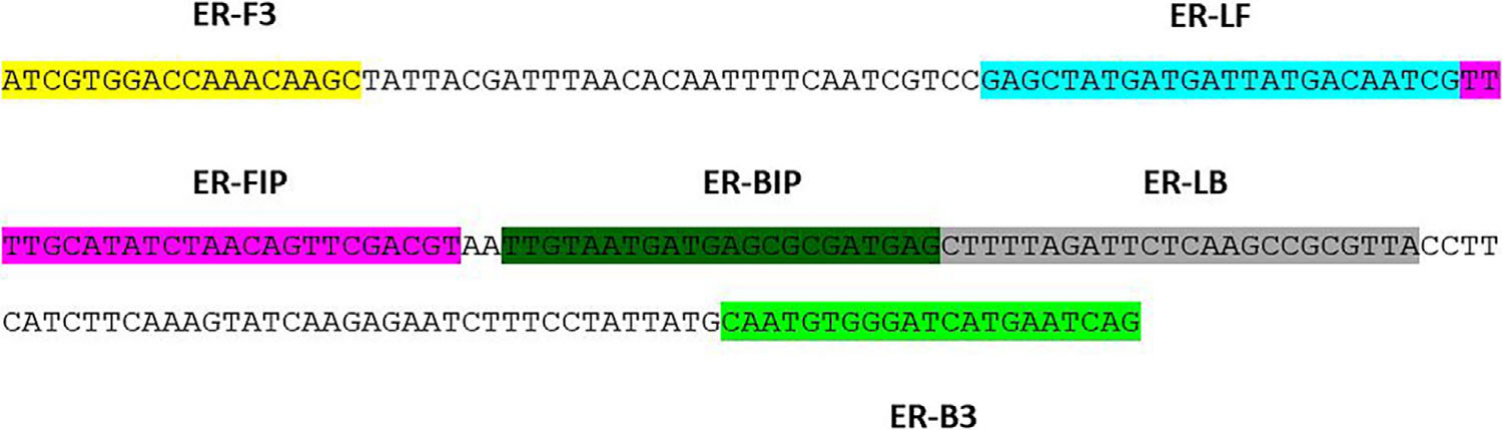
Sequences of LAMP and PCR primers from the putative polypeptide gene, highlighting the relative positions of PCR primers (ER‐F3 and ER‐B3) and LAMP primers, are colour‐coded.

The published primers (Yamazaki et al. 2014) targeting a putative polypeptide gene involved in capsular polysaccharide synthesis, which demonstrate high conservation and specificity for *E. rhusiopathiae*, were used in this study. All the primers were synthesised by Sigma‐Aldrich, Australia. The relative position of the LAMP primers on the target DNA sequence is presented in Figure 1 and is colour‐coded for clarity.

### 2.4 PCR Optimisation and Agarose Gel Electrophoresis

A gradient PCR (48.5 to 65°C), using DNA from the *E. rhusiopathiae* vaccine strain, was initially performed to optimise the annealing temperature for subsequent PCR assays. PCRs were carried out using GoTaq Green Master Mix (Promega, Australia), as previously described (Poussard et al. 2024), together with the published F3 and B3 primers. These PCR reactions were subsequently subjected to agarose gel electrophoresis to assess the efficacy and specificity of target DNA amplification. The optimal annealing temperature was determined based on the results obtained from agarose gel electrophoresis and was subsequently applied in the following PCR experiments.

The final thermocycling conditions were 95°C for 3 min, followed by 28 cycles of 95°C for 30 s, 55°C for 30 s, and 72°C for 30 s, with a final extension at 72°C for 5 min. PCR products were analysed on 1.5% agarose gels to confirm amplification.

### 2.5 Loop‐Mediated Isothermal Amplification

LAMP assays were performed using the WarmStart Colorimetric LAMP Master Mix (New England Biolabs, Australia) and the published primer set, following the approach described previously (Garner et al. 2022). Reactions were incubated at 65°C for 60 min, and the successful amplification of *E. rhusiopathiae* was indicated by a visible colour change from red to yellow.

### 2.6 Evaluation of Detection Limits and Specificity of Developed Assays

To assess the specificity of both PCR and LAMP assays, DNA was extracted from 13 distinct bacterial species. Additionally, the limit of detection of each assay was determined using 10‐fold serial dilutions of 1 ng/µL of DNA extracted from the *E. rhusiopathiae* vaccine strain, up to a concentration of 10^−6^ ng/µL dilution.

### 2.7 Comparison of PCR and LAMP as Detection Methods

Microbiological culture was used as the reference test for evaluating diagnostic performance. The sensitivity and specificity of the LAMP and PCR assays were compared against culture results using a 2 × 2 contingency table analysis (http://www.medcalc.org/calc/diagnostic_test.php).

## 3 Results

### 3.1 Detection of *E. rhusiopathiae* Using PCR and LAMP

When subjected to PCR, the *E. rhusiopathiae* positive control yielded a DNA amplicon of the expected size (207 bp) in agarose gel electrophoresis, while the negative control did not yield any DNA bands. Similarly, in LAMP assay, negative control yielded negative results (with no change in reaction colour, i.e., remaining red), whereas the *E. rhusiopathiae* positive control produced positive results (indicated by a colour change to yellow, indicating the amplification of the target DNA). The specificity of PCR and LAMP was assessed by subjecting them to DNA from 13 distinct bacterial strains. In both assays, only *E. rhusiopathiae* was detected, with no positive results observed for unrelated bacterial strains. The outcomes of the specificity test are presented in Table 1.

**TABLE 1:** PCR and LAMP results for the assessment of unrelated bacterial strains.

Bacterium	LAMP	PCR
*Salmonella typhimurium*	−	−
*Streptococcus equi* (subsp. *zooepidemicus*)	−	−
*Staphylococcus aureus*	−	−
*Escherichia coli*	−	−
*Klebsiella pneumoniae*	−	−
*Staphylococcus* sp.	−	−
*Pseudomonas aeruginosa*	−	−
*Salmonella* *enteritidis*	−	−
*Enterobacter cloacae*	−	−
*Staphylococcus intermedius*	−	−
*Pasteurella multocida*	−	−
*Mycoplasma synoviae*	−	−
*Mycoplasma gallisepticum*	−	−
*Erysipelothrix rhusiopathiae*	+	+
*Negative control*	−	−

*Note*: “+” indicates a positive detection, and “—” indicates a negative result.

### 3.2 The Limit of Detection for PCR and LAMP

**FIGURE 2:**
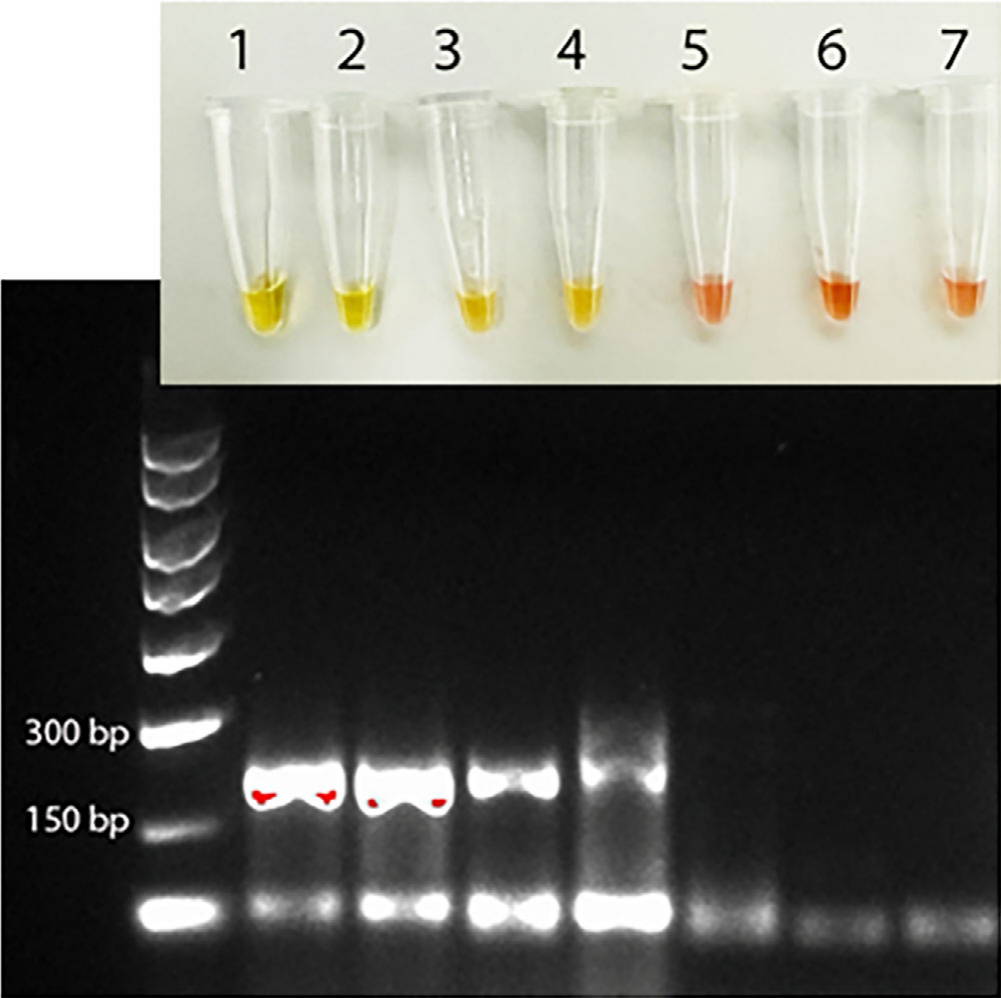
Detection limit of PCR and LAMP using serial 10‐fold dilutions of DNA template. Lane 1: 1 ng of DNA, Lane 2: 10^−1 ^ng, Lane 3: 10^−2 ^ng, Lane 4: 10^−3 ^ng, Lane 5: 10^−4 ^ng, Lane 6: 10^−5 ^ng and Lane 7: 10^−6 ^ng of DNA.

PCR and LAMP assays performed using serial 10‐fold dilutions of DNA extracted from the vaccine strain indicated that both were capable of detecting as little as 10^−3^ ng of extracted DNA in the reaction mixture (Figure 2).

### 3.3 Detection of Erysipelothrix in Clinical Samples Using PCR and LAMP Assay

**FIGURE 3:**
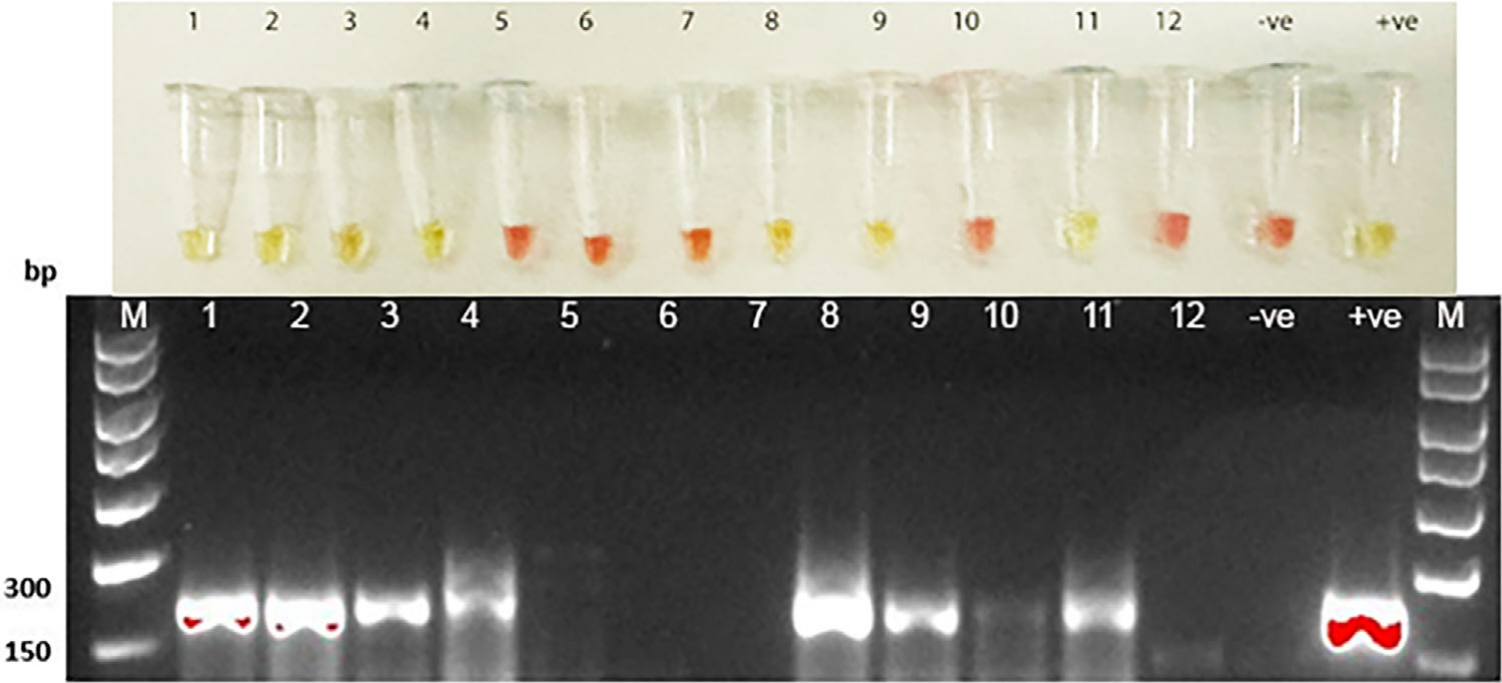
LAMP and PCR results for 12 clinical swabs from different organs collected from suspected chickens.

A total of 52 clinical samples collected during active poultry outbreaks were tested using PCR and LAMP. Both PCR and LAMP assays were successful in detecting *E. rhusiopathiae* in liver, spleen, and bone marrow samples collected from suspected chickens in the field. The same positive outcome was observed for the positive control, which consisted of the *E. rhusiopathiae* vaccine strain. Conversely, the negative control (dH_2_O) produced consistently negative results in both PCR and LAMP assays (Figure 3).

Table 2 presents a summary of the results obtained from PCR and LAMP assays for clinical organ samples collected from suspected chickens with potential *Erysipelothrix* spp. infections. The detection outcomes are further compared with control samples, using two different DNA extraction procedures, the commercial kit and the HotShot (alkaline lysis) method. The results indicated that samples 1, 3, 4, 8, 9, and 11, originating from the liver and spleen, consistently produced positive results in both PCR and LAMP assays, indicating the presence of *E. rhusiopathiae*. However, samples 5, 6, 7, 10, and 12, which were bone marrow samples, produced negative results in culture and LAMP assay when DNA extracted using commercial kit or alkaline lysis method, indicating the absence of the pathogen in these particular samples, except for a single instance in PCR where sample 10 DNA extracted using the commercial kit produced a faint but positive DNA band. The results for positive and negative controls confirm the effectiveness of both PCR and LAMP assays and demonstrate their reliability in identifying *E. rhusiopathiae* in the test.

**TABLE 2:** PCR and LAMP results for clinical organ samples collected from suspected chickens for the presence of *Erysipelothrix* spp., as well as control samples using two DNA extraction methods.

			PCR		LAMP	
Sample No.	Source	Bacterial culture	Commercial kit	HotShot	Commercial kit	HotShot
1	Liver	+	+	+	+	+
2	Bone marrow	+	+	+	+	+
3	Liver	+	+	+	+	+
4	Spleen	+	+	+	+	+
5	Bone marrow	−	−	−	−	−
6	Bone marrow	−	−	−	−	−
7	Bone marrow	−	−	−	−	−
8	Liver	+	+	+	+	+
9	Liver	+	+	+	+	+
10	Bone marrow	−	+	−	−	−
11	Spleen	+	+	+	+	+
12	Bone marrow	−	−	−	−	−
Positive control	ER vaccine strain	NT	+	+	+	+
Negative control	dH_2_O	NT	−	−	−	−

*Note*: “+” indicates a positive detection, and “—” indicates a negative result.

Abbreviation: NT, not tested.

The results from testing clinical samples indicated that these molecular tests can effectively detect *E. rhusiopathiae* in different organ samples, especially when testing bone marrow samples with high fat content.

A total of 40 cloacal samples were also collected from a turkey flock and were subjected to DNA extraction using both the alkaline lysis and commercial kit methods and PCR and LAMP assays were conducted on these DNA samples. All 40 samples showed negative results in both the PCR and LAMP assays, except for the positive control, which effectively confirms the absence of *E. rhusiopathiae* in the collected samples. A summary of all 52 clinical samples used for diagnostic evaluation is presented in Table 3. The dataset included 12 organ swabs collected from chickens during an active erysipelas outbreak and 40 cloacal swabs collected from a turkey flock with a history of erysipelas.

**TABLE 3:** Summary of 52 clinical samples used for the evaluation of PCR and LAMP assays for detection of *Erysipelothrix rhusiopathiae*.

Sample type	Number tested	Culture positive	Culture negative	PCR positive	PCR negative	LAMP positive	LAMP negative
Chicken organ swabs	12	7	5	8	4	7	5
Turkey cloacal swabs	40	0	40	0	40	0	40
Total	52	7	45	8	44	7	45

Given that all clinical swab samples were subjected to DNA extraction using the two distinct methods, alkaline lysis and commercial kit methods, it is noteworthy that the results obtained from both DNA preparations consistently demonstrated similarity in both PCR and LAMP assays, resulting in nearly identical outcomes except for sample 10 using commercial DNA extraction kit and PCR. If the bacteriological culture is considered the gold standard test, this PCR result may be regarded a false positive.

### 3.4 Comparative Analysis of PCR and LAMP Specificity and Sensitivity

Analytical sensitivity and specificity assessments were conducted using DNA samples extracted from the *E. rhusiopathiae* vaccine strain and a diverse selection of bacterial cultures obtained from unrelated strains. Given that these samples, consisting of bacterial cultures, demonstrated the minimal presence of inhibitory factors, both PCR and LAMP assays exhibited robust performance. Specifically, both assays successfully detected *E. rhusiopathiae* in the samples from the vaccine strain and produced negative results when tested with unrelated bacterial isolates. Therefore, the assays demonstrated 100% analytical sensitivity and specificity in this controlled experimental setting.

### 3.5 Evaluation of Clinical Sensitivity and Specificity for PCR and LAMP Assay

The performance of PCR and LAMP tests in detecting *E. rhusiopathiae* in 52 clinical samples including swabs from various organs and cloacal swabs was assessed using a 2 × 2 contingency table. Two DNA extraction methods, alkaline lysis method and the commercial kit, were used in conjunction with each sample. Microbiological culture was considered the gold standard for detecting the pathogen. The results indicate that both PCR and LAMP tests showed 100% clinical sensitivity in all scenarios, regardless of the DNA extraction method used. PCR demonstrated a specificity of 97.78% when using the commercial kit, while LAMP maintained a 100% specificity in all cases. The accuracy of PCR in detecting *E. rhusiopathiae* in clinical samples was slightly lower (98.08%) when compared with LAMP assay (100%) (Table 4). Overall, these results demonstrate that both PCR and LAMP are able to accurately detect *E. rhusiopathiae* in clinical samples.

**TABLE 4:** Clinical sensitivity, specificity, and accuracy of PCR and LAMP assays using different DNA extraction methods, with bacterial culture as the reference method.

Assay	DNA extraction method	True positive	True negative	False positive	False negative	Sensitivity (%)	Specificity (%)	Accuracy (%)
PCR	Commercial kit	7	44	1	0	100	97.78	98.08
PCR	HotShot	7	45	0	0	100	100	100
LAMP	Commercial kit	7	45	0	0	100	100	100
LAMP	HotShot	7	45	0	0	100	100	100

## 4 Discussion

This study primarily aimed to develop a field‐deployable LAMP‐based assay for detecting *E. rhusiopathiae*, in conjunction with a rapid DNA extraction procedure that can be used in field without needing any sophisticated laboratory equipment. The isothermal nature of LAMP‐based assays negates the need for thermal cycling, which makes these assays suited for use in field. However, despite being described for the first time in 2000, the use of LAMP‐based diagnostics in field conditions has been restricted, in part, due to the lack of associated field‐based template preparation methods and product detection formats (Njiru 2012). Therefore, in addition to developing the LAMP assay, the study also aimed to evaluate its efficacy against a conventional diagnostic test like PCR, when applied to clinical samples in conjunction with a rapid field‐based DNA extraction procedure. The results indicate that the accuracy of LAMP‐based diagnosis (100%) was equivalent to traditional microbiological culture and comparable to PCR for detecting *E. rhusiopathiae* in these clinical samples. A single discordant result was observed with PCR when DNA extracted using the commercial kit was tested, resulting in a marginal difference in diagnostic accuracy between the two assays. These results also indicate the efficacy of the rapid DNA extraction procedure used in this study in capturing DNA, in conjunction with the LAMP‐based assay. Clinical specimens frequently have minimal DNA from target pathogens, and any loss of DNA material during the extraction procedure can result in false negative results, reducing the efficacy of the associated diagnostic assay. The DNA extraction procedure used in this study also did not rely on any specialised laboratory equipment, which makes it suitable for deployment on farms for routine surveillance (Truett et al. 2000).

Importantly, this workflow was evaluated using 52 clinical samples collected during active poultry outbreaks, demonstrating its performance using clinical samples collected during active poultry outbreaks. Our findings also indicate that LAMP‐based assays can provide cost‐effective alternatives to conventional laboratory‐based diagnostics such as PCR. In colorimetric LAMPs in particular, the ability to infer results by simple visual observations of a colour change negates the need for any additional expenses unlike PCR where agarose gel electrophoresis is frequently needed to visualise successful DNA amplification and infer results.

Furthermore, in terms of costs involved in routine surveillance and diagnosis, the need for a cold chain represents a considerable expense during the transportation and storage of molecular diagnostic reagents (Njiru 2012). For instance, transporting these reagents from diagnostic laboratory to the field requires the use of dry ice and incurs substantial transportation charges. This still applies to LAMP‐based assays, and therefore the viability of lyophilising LAMP reagents needs to be explored in future to eliminate the need for a cold chain during transportation and storage, which would further improve the utility of LAMP‐based assays in diverse settings, providing a valuable advantage in scenarios where maintaining a constant cold chain might be logistically challenging or financially burdensome (Thekisoe et al. 2009). This study demonstrates the efficacy, cost‐effectiveness, and potential for on‐farm deployment of a field‐ready LAMP assay for the detection of *E. rhusiopathiae*, while also addressing challenges related to field application.

The versatility of the LAMP assay demonstrated in this study is an important factor, particularly in the ability to perform consistently across a range of different sample types. From swab samples collected from various organs and tissues with varying amounts of blood, fat, and other components, to cloacal swabs containing faecal materials and bacterial cultures, the LAMP assay demonstrated a satisfactory level of adaptability and reliability. This broad applicability is essential in real‐world scenarios where samples vary in origin and composition.

However, further exploration of variations in sample characteristics, such as the presence of blood, fat, and other potential inhibitory components, across different poultry farms and environmental conditions could provide insights into the LAMP assay's adaptability and reliability in diverse real‐world scenarios. Future work should include testing the workflow on environmental samples and on other animal species to further expand its applicability beyond poultry outbreaks.

The consistent performance across the diverse sample types shows the LAMP assay potential as a robust diagnostic tool, capable of providing accurate and timely results in a range of clinical and environmental settings.

Additionally, it is important to note that the alkaline lysis method, used for DNA extraction, also exhibited good performance, providing acceptable quality DNA samples for both LAMP and PCR assays to perform effectively. The high similarity results between bacterial culture and LAMP assay highlights the efficacy and reliability of the alkaline lysis method, as a viable alternative to commercial kits for DNA extraction in field conditions without specialised laboratory equipment. Similar results between PCR and LAMP and their concordance with microbiological culture findings are indicative of the efficacy of these molecular methods.

In addressing key economic considerations for the adoption of the LAMP assay in poultry farms, several factors should be considered. The LAMP assay, with a turnaround time of 60 min, stands out in producing results in a minimal timeframe. This processing time, when compared to PCR, has the advantage of delivering relatively quicker results in contrast to more traditional diagnostic methods.

Furthermore, the LAMP assay's suitability for field applications is a significant economic factor, requiring minimal instrumentation. Unlike PCR, LAMP does not demand the same level of technical expertise for setup and result interpretation, contributing to its practicality and cost‐effectiveness in resource‐limited settings. These aspects collectively position the LAMP assay as an economically viable and user‐friendly option for on‐site pathogen detection, opening avenues for extensive integration across diverse environments.

However, considering the potential impact on the overall diagnostic workflow, it would be valuable to investigate the integration of the alkaline lysis method into routine on‐farm diagnostic practices and assess its scalability and robustness under different field conditions.

The alkaline lysis method appeared to be practical and cost‐effective, making it an appealing choice for field‐based DNA extraction method. This aligns with our study's goal of developing a user‐friendly, farm‐ready solution for *E. rhusiopathiae* detection. These findings represent a significant advance towards the development of an accurate in‐field diagnostic method capable of detecting targeted pathogens, including *E. rhusiopathiae*, in poultry farms.

## Author Contributions

Seyed Ali Ghorashi, Sameer D. Pant, Jiongrui Huang, and Peter Scott: conceptualisation. Seyed Ali Ghorashi and Sameer D. Pant: formal analysis. Seyed Ali Ghorashi, Sameer D. Pant, and Jiongrui Huang: investigation. Seyed Ali Ghorashi, Sameer D. Pant, Jiongrui Huang, and Peter Scott: methodology. Jiongrui Huang and Peter Scott: resources. Seyed Ali Ghorashi: supervision. Seyed Ali Ghorashi and Sameer D. Pant: validation. Seyed Ali Ghorashi: writing – original draft. Seyed Ali Ghorashi, Sameer D. Pant, Jiongrui Huang, and Peter Scott: writing – review and editing.

## Funding

This work was financially supported by the Poultry Hub Australia under grant number 21‐308 and School of Agricultural, Environmental and Veterinary Sciences at Charles Sturt University.

## Ethics Statement

The study was performed under Animal Care and Ethics approval (Protocol A21381) at Charles Sturt University.

## Conflicts of Interest

The authors declare no conflicts of interest.

## Data Availability

The data supporting the findings of this study are available from the corresponding author upon reasonable request.
